# Mens Sana in Corpore Sano

**Published:** 1917-05-12

**Authors:** 


The Hospital

The Workers' Newspaper of Administrative Medicine and Institutional Life, Administration, National Insurance and Health.

No. 1614, Vol. LXII. SATURDAY, MAY 12, 1917. PRICE ONE PENNY

### MENS SANA IN CORPORE SANO.

It is rare to find in any document, still rarer to find in an official document, as much good sense and good reasoning as is to be found in the final Report just issued by the Departmental Committee on Juvenile Education, etc. The effect of the war upon children and young persons, or, as the Report ungrammatically calls them, juveniles, has been in many ways detrimental and pernicious. It has greatly diminished attendance at schools, especially evening schools; occupations formerly carried on by boys, and unsuitable for girls, have been transferred to girls. Parental control has been relaxed by the absence of fathers from homes; wages have been suddenly and greatly raised; even the ordinary discipline of the workshop has given way; excessive hours of labour have overtaxed the energies of the young; many have taken advantage of the extraordinary demand for juvenile labour to change even more frequently than usual from one blind-alley employment to another. In consequence of all these conditions there has been a noticeable alteration in behaviour and morality.

The remedy suggested is "nothing less than a complete change of temper and outlook on the part of the people of this country as to what they mean, through the forces of industry and society, to make of their boys and girls." We cordially agree. "Can the conception of a juvenile as primarily a little wage-earner be replaced by the conception of the juvenile as primarily the workman and citizen in training?" We would add, Can the conception of a cultured person as a person who can read a Greek play be replaced by the conception of the cultured person as a person who can form, upon the ample general information that he possesses, rational judgments on matters outside of his immediate occupation?

Evening classes, while they are not altogether condemned, and while they receive credit for the benefits they have conferred upon "many generations of children, fortunate enough in their parents, or in their employers, or in their own physical strength and force of character, to take advantage of them, are yet shown to be insufficient. "We do not want any more of the waste and overstrain involved in the teaching of tired pupils by tired teachers." Again, we cordially agree, and so we do with the following:—"We are clear that the business of the classes is to do what they can in making a reasonable human being and a citizen, and that, if they do this, they will help to make a competent workman also." Yes, and training directed to the same immediate end will help to make a competent physician, or lawyer, or merchant also. "There is not the slightest doubt that from a physical point of view the break in a child's life when he leaves school [at fourteen] comes just at the moment when the disturbance of puberty brings him most in need of skilled and systematic direction. And this is just the moment when he has to face the new strain of employment." Again, we appreciate the wisdom and the grip of realities. It is all pretty obvious, no doubt; but it is just the obvious that is most overlooked and ignored in our present system of education, at any rate in our public schools and universities.

"Evening Schools, over and above their educational functions, have in the past done valuable service in keeping children off the streets. It would be a regrettable set-off against the educational advantages if the transference of Continuation Classes to the day-time were to remove one of the few facilities available in crowded towns for a wise employment of the evening hours. . . . The school buildings at least, perhaps not the main, under voluntary supervision, should continue to be at the service of the juveniles, for whom the evening hours will in future be to a greater extent available as hours for wholesome recreation. In the summer, recreation should as far as possible be in the open air. The evenings will in future be longer, if the operation of the Daylight Saving Act, which we regard as one of the most beneficial of the by-products of the war, is continued in times of peace. There will be games, and gardening, and scout-craft. It is the winter which will demand resources other than the poor alternatives offered by the inclement streets, the gambling pitch under the railway arch, and the garish entertainments, which appear to be all that the low-grade theatres and picture palaces care to provide. The Continuation School, like the Day School, ought to become a centre for the self-directed activities of the pupils, as well as for those imposed upon them, and its buildings should serve as a home for innumerable clubs, debates, study circles, concerts, and other forms of social gathering. In the evening, too, the serious instruction of the day-time may be supplemented by voluntary classes in recreative subjects for those who desire them." Physical training, too, is advocated, both for the prevention of physical defects and deformities and to develop bodily control, graceful movements, and a sense of rhythm.

If these aspirations lead to their own realisation in actual practice, and, since we have for the first time in our history a Minister of Education who knows something about education, it seems likely that they will be, the chances that future generations of our race will have healthy minds in healthy bodies will be indefinitely increased. The matter is primarily one of education; but it is secondarily, and at no distant remove, one of health, both bodily and mental, and therefore it is that we welcome this Report of the Departmental Committee.

